# Combined aza-Michael and radical photopolymerization reactions for enhanced mechanical properties of 3D printed shape memory polymers†

**DOI:** 10.1039/d2ra05404c

**Published:** 2022-10-25

**Authors:** Lucile Halbardier, Emile Goldbach, Céline Croutxé-Barghorn, Anne-Sophie Schuller, Xavier Allonas

**Affiliations:** Laboratoire de Photochimie et d'Ingénierie Macromoléculaires, Institut Jean Baptiste Donnet 3b rue Alfred Werner 68093 Mulhouse Cedex France celine.croutxe-barghorn@uha.fr

## Abstract

3D printed shape memory polymers (SMP) were formed by combining aza-Michael addition and light initiated radical polymerization. Amine consumption and acrylate conversion were monitored by ^1^H-NMR and Fourier transform infrared spectroscopies. Dynamic mechanical analysis and cyclic thermomechanical tensile tests enabled direct observation of the polymer network changes. Increased homogeneity of the 3D network and enhanced SMP properties were achieved after the reaction between residual acrylate functions trapped in the vitrified medium with the secondary amines formed during the process. This allows the fabrication of shape memory objects by 3D printing.

## Abstract 2

A shape memory polymer formed from combined aza-Michael addition and radical photopolymerization.

## Graphical abstract

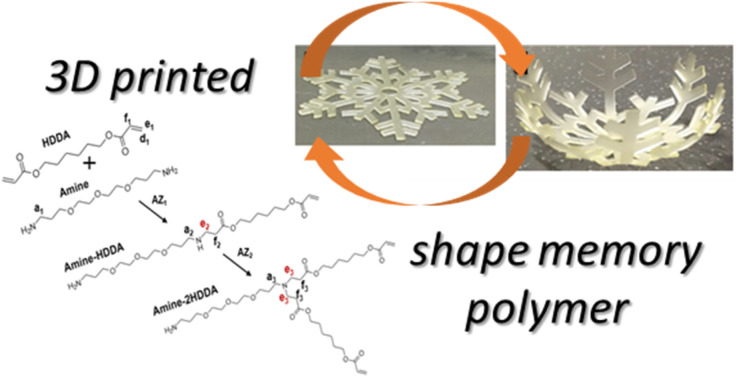

Among the various shape memory polymers (SMP),^1^ those exhibiting changes in shape under temperature are those widely implemented in industry.^2^ SMP can be made from different kinds of polymers (polyurethanes,^3^ epoxys,^4,5^ acrylates^6–10^) depending on the application. Used in the biomedical field^11^ for implants, drug delivery systems,^12,13^ stents or vascular grafts,^14,15^ SMP are also implemented in the aerospace industry for the deployment of structure^16–19^ and in the robotic field.^20,21^ They are generally made *via* molding, which is a high cost process due to the fabrication of molds, and often exhibit limited shape complexity. Today, a large part of these materials are made by 3D printing, through fused deposition molding (FDM),^22^ direct ink writing^23^ (DIW), stereolithography (SLA)^24^ or digital light processing (DLP).^6^

For SLA and DLP processes, acrylate monomers are used because of their high reactivity and their commercial availability. Although radical photopolymerization allows a fast growth of the polymer material, it is known to yield premature vitrification and an internal network heterogeneity. Consequently, the final material has limited shape memory properties. Such a material, which exhibits a high crosslink density leads under evaluation of its SMP properties to important internal stress during its stretching.^25,26^ When cooled down at *T* < *T*_g_ and upon maintained strain, it undergoes additional intramolecular rearrangements to reach a stable temporary form, a fact which decreases the final fixation ratio. In addition, the high crosslink density also limits the chain mobility during the recovery of the original shape at *T* > *T*_g_, therefore decreasing the recovery ratio.

To tackle the drawbacks, several strategies have been considered. They mainly consist in reducing the crosslink density of the polymer network. For example, the synthesis of high molar weight diacrylate prepolymers based on polycaprolactone and polypentadecalactone was reported.^7^ Mixed with a weak amount of crosslinking agent, it resulted in a polymer network exhibiting two different melting temperatures, leading thus to a reversible programmable SMP. The use of *tert*-butylacrylate combined with 10% of 1,6-hexanedioldiacrylate was also considered.^8^ Another approach to limit the number of crosslinks is to create physical crosslinks like hydrogen bonds. These latter can stabilize the temporary shape when the material is stretched and cooled below its *T*_g_. Upon heating, these physical crosslinks are broken, thereby increasing polymer chains mobility. This facilitates the return to the original shape. Following this approach, different ratios of high molecular weight urethane diacrylate and *tert*-butylacrylate have been investigated successfully and their SMP properties assessed.^9^ Combination of monofunctional methacrylates and a multifunctional photoinitiator seems also particularly attractive for SMP generation.^10^ The slightly crosslinked polymer network was stabilized by hydrogen bonds created between the hydroxyl function from hydroxyethylmethacylate (HEMA) and the carbonyl groups of the acrylate functions. As can be seen, these different approaches, although successful, are focused on the use of large amount of monofunctional monomers, which merely leads to low cross-linking density and therefore, reduced mechanical properties.

In this paper, the chemical approach consists in the *in situ* formation of a branched poly(amino ester) prepolymer at room temperature and without preliminary synthesis, that is subsequently photopolymerized under a 3D printing step. An aza-Michael addition reaction (AZ_1_) first takes place between a primary diamine and an acrylate in large excess to generate a secondary amine monoadduct noted AMS (Fig. 1a). Then the resulting prepolymer is photopolymerized, in presence of a photoinitiator, under subsequent exposure in the 3D printer. After full completion of the 3D building process and light processed post-curing, a second and slow nucleophilic aza-Michael addition (AZ_2_) starts in the dark.^27–29^ It results in a further cross-linking process between the generated secondary amines AMS and unreacted acrylates trapped in the vitrified medium.^27^ This yields a tertiary amine diadduct (ADT) (Fig. 1b). This reaction leads to a more homogeneous network as shown by DMA. The resulting polymer exhibits shape memory effect with high degree of fixation of the temporary shape.

**Fig. 1:**
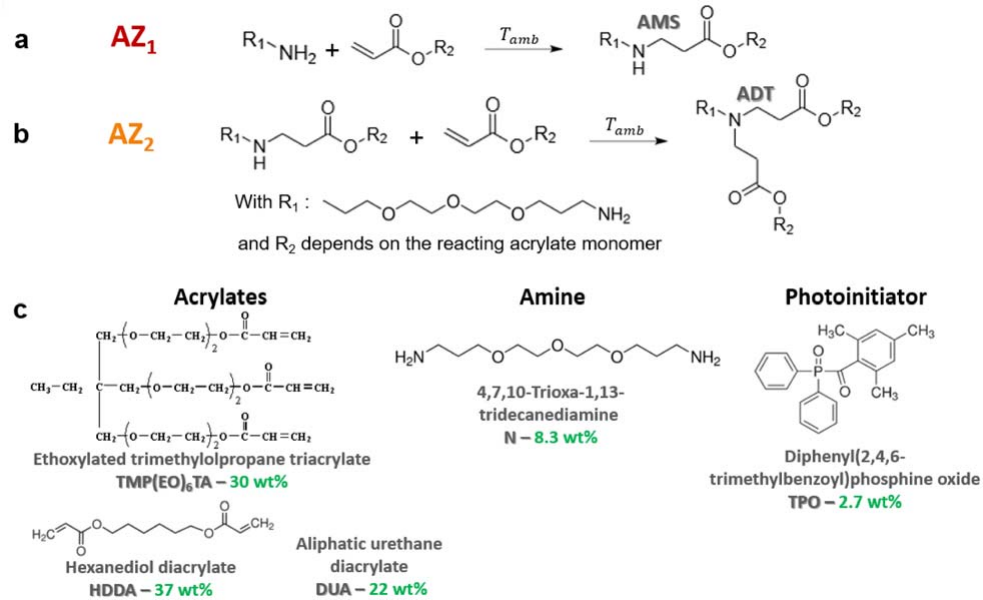
(a) First aza-Michael addition (AZ_1_) of a primary amine on the β-carbon of the α,β-unsaturated carbonyl to form AMS, (b) second aza-Michael addition (AZ_2_) of a secondary amine on β-carbon of the α,β-unsaturated carbonyl leading to the formation of ADT and (c) structure and weight percentage of the different components in the formulation.

The formulation investigated in this paper consists in a mixture of 4,7,10-trioxa-1,13-tridecanediamine (N, 8.3 wt%, 220 g mol^−1^, from TCI), an ethoxylated trimethylolpropane triacrylate (TMP(EO)_6_TA, 30 wt%, 554 g mol^−1^, SR499, from Sartomer), an aliphatic urethane diacrylate (DUA, 22 wt%, 2200 g mol^−1^, CN981, from Sartomer) and 1-6-hexanedioldiacrylate (HDDA, 27 wt%, 226 g mol^−1^, from Sartomer). TMP(EO)_6_TA was used as flexible crosslinker and HDDA allows to increase the reactivity during 3D printing (Fig. 1c).

The molar ratio between amine and acrylate functions has an impact on the overall formulation reactivity and the beginning of AZ_2_. Therefore, a ratio of 1/7 of amine/acrylate functions was chosen for this study after optimization. Using this ratio, the amount of AMS species generated in the first step would induce a maximal consumption of 14% of the acrylate functions. Then, completion of all the AMS adducts would additionally consume a maximal of 14% of acrylate groups. Therefore, these two aza-Michael additions induce a maximal consumption of 28% of acrylate double bonds.

The reaction of the acrylate double bonds leads to the creation of new bonding between methylene and amine, on β-position of the carbonyl group of acrylates. This could be monitored by ^1^H-NMR measuring the appearance of new chemical shift between 2 and 3 ppm for both adducts resulting from AZ_1_ and AZ_2_, at *δ*_CH_2_–NH_ = *δ*_*e*_2__ = 2.87–2.81 ppm and *δ*_CH_2_–N_ = *δ*_*e*_3__ = 2.76–2.69 ppm, respectively (ESI Fig. S1 and Table S1†).

The acrylate consumption during the aza-Michael addition was also estimated by Fourier transform infrared spectroscopy in attenuated total reflection mode (ATR-FTIR, see ESI†). Changes in the generated and consumed species with time are reported in Fig. 2. Concerning AZ_1_, the reaction takes place during the first 120 minutes and levels off to a value of 16 ± 2% that is close to the theoretical value of 14%. During this period, the amount of acrylate groups consumed reaches the same value of 16 ± 2%. During this reaction time, the generated ADT does not react with the acrylate double bonds due to their steric hindrance.^28,30–32^ Focusing now on AZ_2_, it could be seen that the amount of AMS drastically decreases from 1 to 7 days. The generated secondary amines AMS further react with acrylate functions to form tertiary amines ADT: the amount of ADT increases progressively to a maximum value of 16 ± 2% after 7 days. The overall acrylate conversion through AZ_1_ and AZ_2_ reaches 26 ± 2%, confirming the almost total consumption of secondary amine. Interestingly, when AZ_1_ takes place, the viscosity of the formulation varies from 100 to 500 mPa s after 4 hours (ESI Fig. S2†). The moderate change in viscosity after AZ_1_ and the important delay between AZ_1_ and AZ_2_ allows to introduce an intermediate photopolymerization step operated with a DLP printer.

**Fig. 2:**
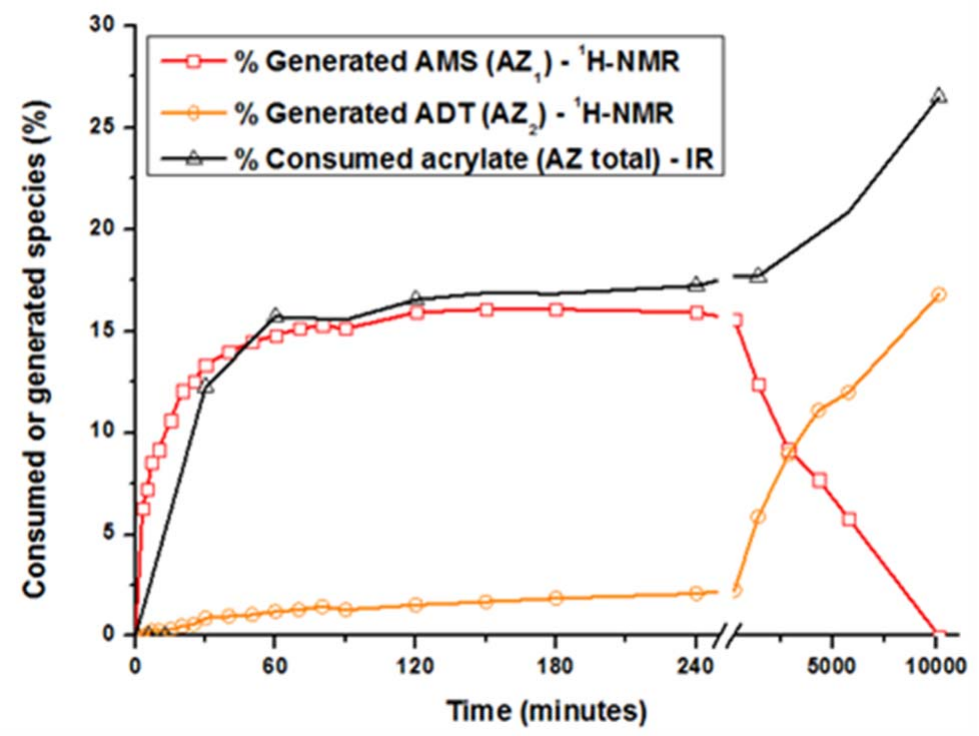
Kinetics of acrylate consumption and generated species (AMS and ADT) during the two aza-Michael addition. The quantitative evaluation was achieved between 0 and 10 000 minutes (7 days) by ATR-FTIR and ^1^H-NMR, respectively.

In order to photopolymerize the resin after AZ_1_ reaction, diphenyl(2,4,6-trimethylbenzoyl)phosphine oxide (TPO) was added as photoinitiator at a load content of 2.7 wt%. Rectangular objects (30 × 5 × 0.3 mm) were printed with an Asiga UV Max printer working at a curing wavelength of 385 nm and an irradiance of 9.8 mW cm^−2^ (details reported in ESI†). After the printing step (noted *hν*), samples were cleaned with ethanol and post-cured 2 min per side with a LED at 385 nm and an irradiation of 70 mW cm^−2^ (noted PC_*hν*_).

The conversion of acrylate double bonds (Conv_C=C_) was estimated by ATR-FTIR with measurements on each side (details reported in ESI, Table S2†). The overall process and the average of the Conv_C=C_ for each step are shown in Fig. 3.

**Fig. 3:**
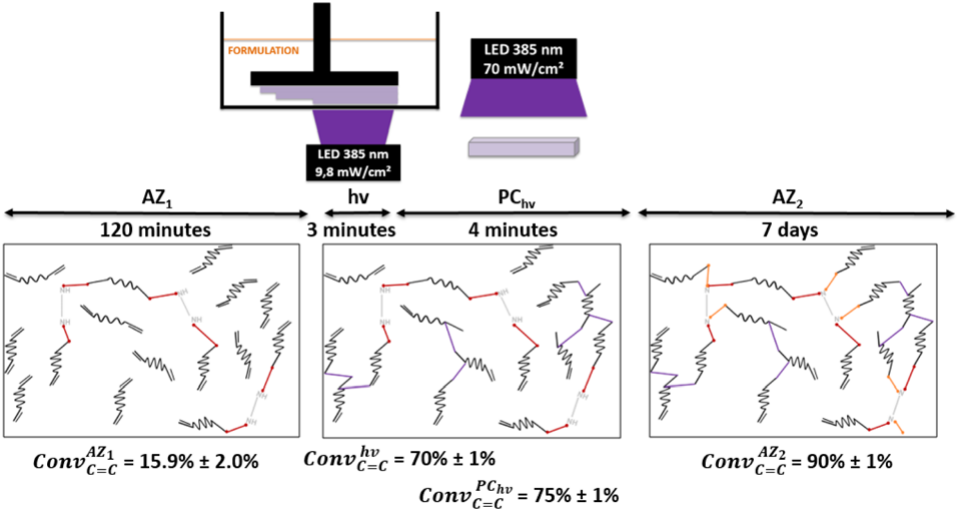
Overall process implemented for the SMP generation including the first aza-Michael addition (AZ_1_), the 3D printing (*hν*), the post-curing (PC_*hν*_) and the second aza-Michael addition (AZ_2_). Acrylate conversion after each step are also reported.

Under irradiation, a fast conversion of acrylate double bonds is obtained and leads to an increase of the viscosity. Rapidly, the radicals and monomers are trapped into the vitrified medium and therefore, the Conv_C=C_ value levels off at 70%. Then, the additional post-curing step under LED at 385 nm and an irradiance of 70 mW cm^−2^ is driven upwards the Conv_C=C_ value to 75%. The aging of the material during 7 days induces an additional conversion increase of 15%, due to the completion of AZ_2_ reaction. This is in full agreement with the results obtained by ^1^H-NMR and FTIR-ATR on the system described in Fig. 2. The 14% of AMS generated during the AZ_1_ were consumed for the ADT generation. The occurring of AZ_2_ allows a total reaction between the AMS and the acrylate functions trapped in the vitrified medium. It could appear quite unusual to observe a dark reaction such as AZ_2_, taking place in a vitrified medium. Therefore, two different samples with (AZ_1_ + *hν* + PC_*hν*_ + AZ_2_) and without (AZ_1_ + *hν* + PC_*hν*_) AZ_2_ completion were compared. The corresponding polymer network properties were measured by dynamic mechanical analysis (DMA). Results are gathered in Table 1. The details of the different parameters and the experimental conditions are reported in ESI.† From these results, it turns out that AZ_2_ reaction leads to an increase of the glass transition temperature (*T*_g_) and the rubber modulus (*E*_r@110°C_) as shown in Table 1. The molecular weights between entanglements (*M*_e_)^33,34^ were estimated from the rubbery modulus (*E*_r_) and the density of the polymer network (*ρ*_polymer_) as evaluated by pycnometry (details reported in ESI†):
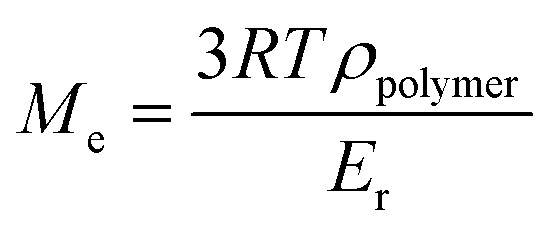


**Table 1:** Characterization of the polymers obtained with and without AZ_2_ reaction from DMA (frequency of 1 Hz, amplitude of 20 μm and temperature range between −70 and 130 °C)^a^

*T*_g_ onset *E*′ (°C)	*E*_r@110°C_ (MPa)	FWHM (°C)	*ρ*_polymer_	*M*_e@110°C_ (g mol^−1^)
−3 ± 2^(a)^	52 ± 4^(a)^	56 ± 2^(a)^	1.13^(a)^	207^(a)^
7 ± 2^(b)^	64 ± 4^(b)^	44 ± 1^(b)^	1.19^(b)^	178^(b)^

a 3D printed samples (30 × 5 × 0.3 mm) were irradiated at 385 nm with an irradiance of 9.8 mW cm^−2^, postcured 2 minutes at 70 mW cm^−2^ and evaluated after the post cure (sample noted (a) AZ_1_ + *hν* + PC_*hν*_) and after AZ_2_ (sample noted (b) AZ_1_ + *hν* + PC_*hν*_ + AZ_2_).

With *M*_e_ = the molar height between entanglements (g mol^−1^), *R* = the molar gas constant (kPa L^−1^ mol^−1^ K^−1^), *T* = the temperature (K), *ρ*_polymer_ = the density of the polymer formed (g mL^−1^), *E*_r_ = the rubber modulus (MPa).

The completion of the aza-Michael reaction is confirmed by the increase in the polymer density and the decrease of *M*_e_. This densification leads to a homogenization of the network generated, as shown by the value of the full width half-maximum (FWHM) of the tan *δ* curve which decreases by 12 °C.

The mechanical properties of the printed object were studied by tensile tests at break by DMA. The evolution of the strain with a stress ramp of 0.5 MPa min^−1^ was recorded at 80 °C (on the rubbery plateau of the storage modulus curve), above the glass transition temperature and reported Fig. 4.

**Fig. 4:**
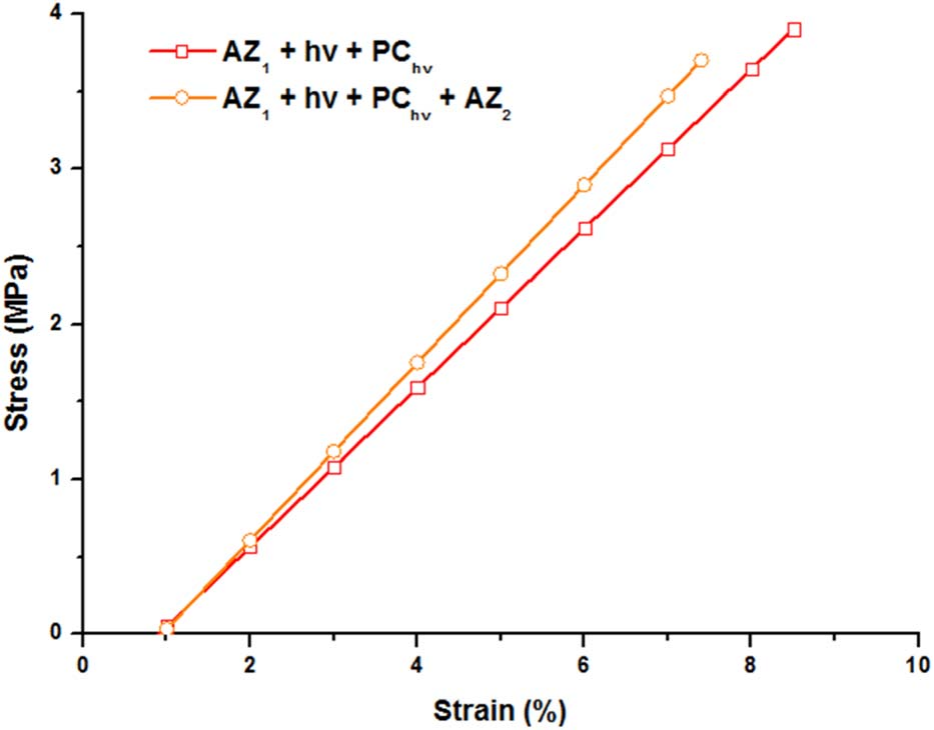
Stress–strain curves of the polymers obtained with a stress ramp of 0.5 MPa min^−1^ at 80 °C. 3D printed samples (30 × 5 × 0.3 mm) were irradiated at 385 nm with an irradiance of 9.8 mW cm^−2^, postcured 2 minutes at 70 mW cm^−2^ and evaluated after the post cure and after AZ_2_.

The 3D printed sample (AZ_1_ + *hν* + PC_*hν*_) exhibits a strain of 8.5% before break for a stress applied of 3.9 MPa. The Young modulus corresponding to the slope of this curve is of 56 MPa. After 7 days and the occurring of AZ_2_, the sample becomes slightly more brittle. A less important stress (3.7 MPa) induces its break and the elongation is limited to 7.6% with a Young modulus of 60 MPa.

Shape memory effect can be studied through the fixation and recovery properties of the material which could be measured during 8 thermocycling tensile tests in controlled stress.^35,36^ The description of the material solicitation by DMA is given in ESI.† This thermomechanical test can be operated after post-curing and after AZ_2_ completion. Fig. 5 shows the result obtained for the printed material (AZ_1_ + *hν* + PC_*hν*_ + AZ_2_). The material heated at 80 °C was stretched with a maximal stress of 3.7 MPa and a maximal deformation of 8%. Then it was cooled down at −50 °C, with a resulting strain of 6.9% at the end of the isotherm. The stress was released and the evolution of the behaviour of the material was recorded at this temperature until the stabilisation of the strain at 6.6%. The final step consisted in an increase of the temperature to 80 °C in order to recover the original step. The final strain was 2%.

**Fig. 5:**
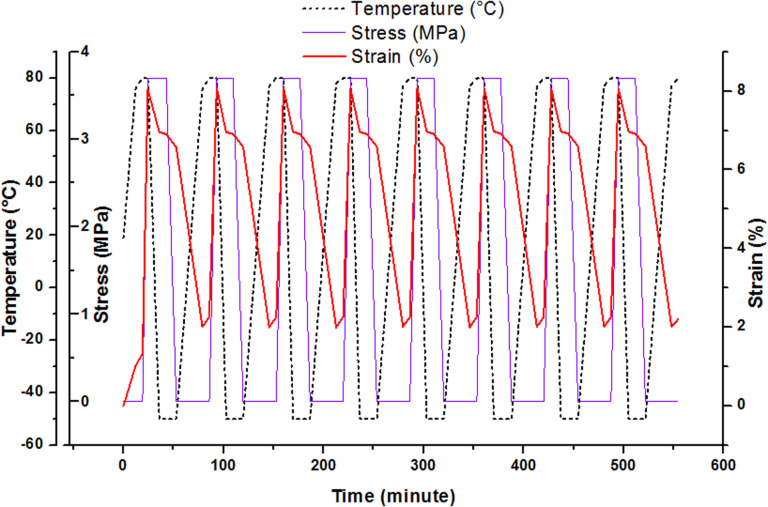
Thermocycling tensile tests for the rectangular printed material (AZ_1_ + *hν* + PC_*hν*_ + AZ_2_) between −50 °C and 80 °C.

The fixation ratio, *R*_f_(*N*), and the recovery ratio, *R*_r_(*N*), were determined from these experiments (Table 2). The first one describes the capacity of the material to be fixed in a temporally shape after cooling and successive thermomechanical tests. The second one, *R*_r_(*N*), corresponds to the capacity of the material to reproduce an equivalent behaviour between each thermocycle.

**Table 2:** Evolution of *R*_f_(*N*) and *R*_r_(*N*) during 8 thermomechanical tensile tests in controlled stress between −50 and 80 °C for the printed objects. Equations to calculate *R*_f_(*N*), and *R*_r_(*N*) are reported in ESI^a^

Cycle (*N*)	*R*_f_(*N*)^(a)^ (%)	*R*_r_(*N*)^(a)^ (%)	*R*_f_(*N*)^(b)^ (%)	*R*_r_(*N*)^(b)^ (%)
*N* = 1	95.5	—	95.5	—
*N* = 2	95.6	99.8	95.1	100.2
*N* = 3	95.5	99.8	95.2	100.0
*N* = 4	95.5	99.7	95.2	99.6
*N* = 5	95.6	99.7	95.2	100.4
*N* = 6	95.3	99.8	95.2	99.8
*N* = 7	96.0	100.0	95.3	100.0
*N* = 8	95.9	99.8	95.2	99.8

a 3D printed samples (30 × 5 × 0.3 mm) were irradiated at 385 nm with an irradiance of 9.8 mW cm^−2^, postcured 2 minutes at 70 mW cm^−2^ and evaluated after the post cure (sample noted (a) AZ_1_ + *hν* + PC_*hν*_) and after AZ_2_ (sample noted (b) AZ_1_ + *hν* + PC_*hν*_ + AZ_2_).

For the both materials, *R*_f_(*N*) and *R*_r_(*N*) are high and reproducible along the 8 thermocycles. These results show that the combination of aza-Michael reactions and photopolymerization is a relevant route to increase the homogeneity of the polymer network, thereby enhancing the SMP properties of a 3D printed object. Less internal stresses are stored into the material when it is stretched and cooled down. It results a high *R*_f_(*N*) of 95%. The mobility of polymer chains during the heating phases is also increased. It contributes to a better material capacity to return to its original shape, which explains the *R*_r_(*N*) of about 100%. Although AZ_2_ leads to an increase of the crosslink density (Table 1), both ratios are not decreased and remain in the same range. The presence of more crosslinks could account for the slightly lower *R*_f_(*N*) values. Nevertheless, the *R*_r_(*N*) is equivalent while the mobility of the chains is reduced. Finally, the homogenisation of the network resulting from the combination of the nucleophilic additions and the photopolymerization allows a more synchronized mobility of the chains upon heating which favours the recovery.

Even though the materials were solicited with variation of temperature during approximatively 9 hours during 8 cycles, almost no changes in the polymer network could be noticed. Therefore, the *R*_f_(*N*) and *R*_r_(*N*) remain constant during the thermomechanical tests.

To demonstrate the feasibility of the overall process and illustrate the shape memory properties of the printed materials, a snowflake with a dimension of 50 × 57 × 0.6 mm was fabricated by 3D printing and postcured under the same conditions than the rectangular objects. After 7 days, the material AZ_1_ + *hν* + PC_*hν*_ + AZ_2_ was heated and stretched at 80 °C above the relaxation temperature (*T*_r_) and cooled down maintaining the stress to obtain the temporary shape. Then, the snowflake was immersed in hot water (80 °C) to recover its original shape. Pictures of the initial, temporary and final shape are shown Fig. 6.

**Fig. 6:**
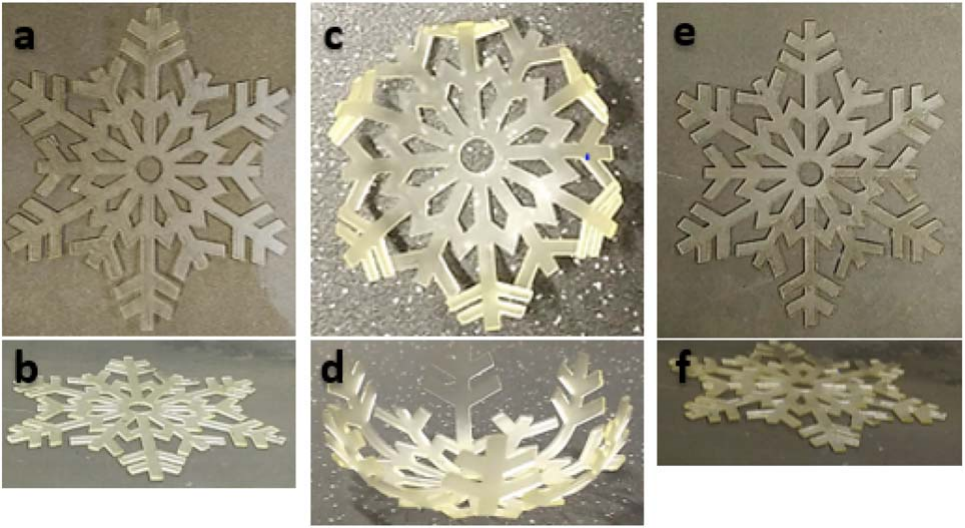
3D printed snowflake AZ_1_ + *hν* + PC_*hν*_ + AZ_2_, (a and b) after 3D printing (@385 nm, 9.8 mW cm^−2^) and post-curing (@385 nm, 70 mW cm^−2^); (c and d) after deforming at 80 °C and cooling; (e and f) after immersion in water at 80 °C and recovery.

In conclusion, the combination of aza-Michael addition and radical photopolymerization has been considered to limit the crosslink density of the polymer network. It affords the *in situ* creation, at room temperature and without preliminary synthesis, of a branched poly(amino ester) prepolymer that is subsequently photopolymerized to achieve 3D objects printed by DLP and exhibiting a shape memory behaviour. The photosensitive resin containing a photoinitiator, primary amines and acrylates in excess has been thoroughly investigated by ^1^H-NMR and ATR-FTIR and the resulting polymer was characterized by DMA. The aza-Michael addition between secondary amines and residual unreacted acrylate functions trapped in the vitrified medium, enhances the polymer structure homogeneity and the resulting mechanical properties. AZ_1_ and AZ_2_ leads to a 3D printed material with high fixation and recovery ratios (95% and 100%, respectively). The high reproducible behaviour of the material during 8 thermocycles highlighted significant thermomechanical performances. This 3-step approach is of particular interest for the shape memory polymer fabricated in 3D printing by DLP.

## Conflicts of interest

There are no conflicts to declare.

## Supplementary Material

RA-012-D2RA05404C-s001
