# Supplementary material for: Combined aza-Michael and radical photopolymerization reactions for enhanced mechanical properties of 3D printed shape memory polymers
Source: RSC Adv. 2022 Oct 25;12(47):30381–5. doi: 10.1039/d2ra05404c (PMC9593170; doi:10.1039/d2ra05404c)
Supplement: RA-012-D2RA05404C-s001 [file RA-012-D2RA05404C-s001.pdf]

Electronic Supplementary Material (ESI) for RSC Advances.
This journal is © The Royal Society of Chemistry 2022

# *Supporting information*

## Combined aza-Michael and radical photopolymerization reactions for enhanced mechanical properties of 3D printed shape memory polymer

Lucile Halbardier, Emile Golbach, Céline Croutxé-Barghorn*, Anne-Sophie Schuller, Xavier Allonas

### Study of the AZM reaction by liquid state $^1$H-NMR spectroscopy

$^1$H-NMR spectra were recorded on a Bruker Advance 400 spectrometer. To allow a full spin relaxation a delay of 8s was used and the spectra were acquired with 16 scans. The chemical shift was referred to the residual proton signal of the solvent $CHCl_3$ at 7.26 ppm for $^1$H. The dissolving in $CDCl_3$, working in a diluted medium, stops the reaction.

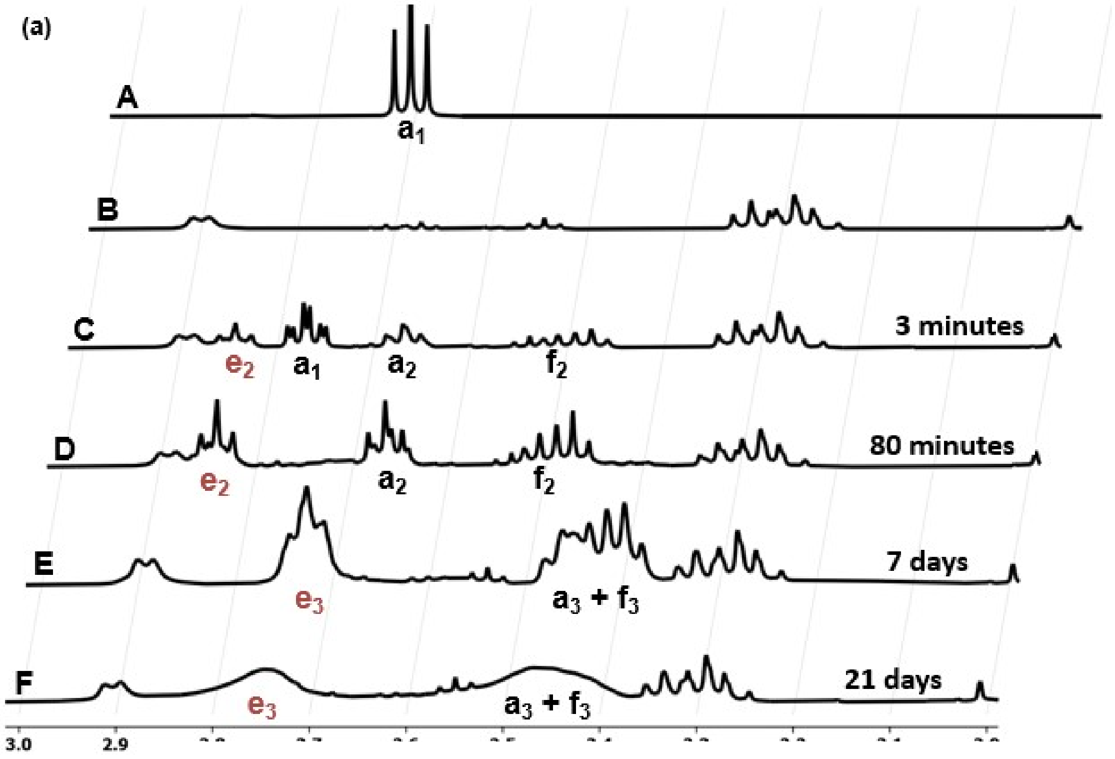


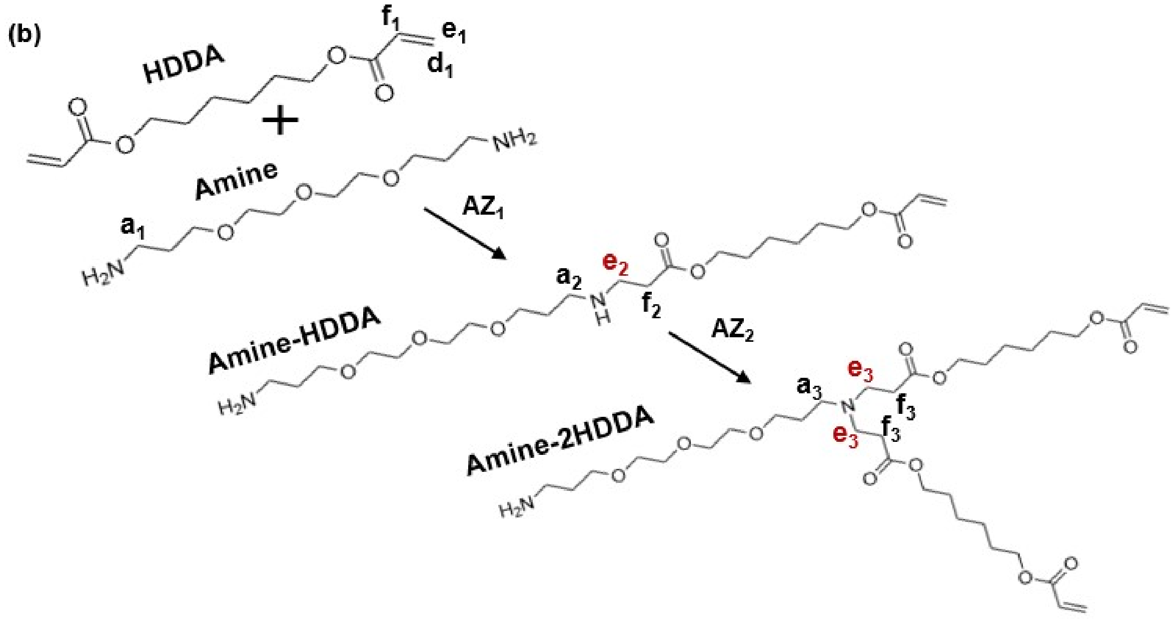


**Figure S1.** (a) $^1$H-NMR spectra recorded for the mixture of the 3 acrylate monomers (A) and for the 4,7,10-trioxa-1,13-

tridecanediamine (B) before mixing all components, and recorded for the global formulation with a 1/7 molar ratio of amine/ acrylate functions after (C) 3 min; (D) 80 min; (E) 7 days; (F) 21 days of reaction. (b) Example of the reaction taking place between 4,7,10-trioxa-1,13-tridecanediamine and hexanediol diacrylate occuring in the middle. The assignments of the protons mentioned on the $^1$H-NMR spectra are also represented on this scheme.

**Estimation of the amount of generated AMS and ADT species during aza-Michael reaction**

Some hypotheses have been made to estimate the percentage of species generated during the aza-Michael reaction:

- The probability of reaction between a specific acrylate and the amine only depends on the amount of acrylate functions into the formulation.
- Each reaction between an amine and an acrylate leads to the same chemical shift of the methylene group $e_2$ at 2.85 ppm and $e_3$ at 2.75 ppm for AMS an ADT, respectively.

The ratio of each acrylate molecules was estimated from the molar weight and the mass of each component in 100g of formulation. The number of $e_2$ and $e_3$ protons for each Michael adducts are reported **Table S1**.

**Table S1**. Ratio of acrylate functions and corresponding amount of $e_2$ and $e_3$ protons for each Michael adducts.

| Components | Ratio of acrylate functions in the formulation (%) | Number of $e_2$ protons (AMS) | Number of $e_3$ protons (ADT) |
|---|---|---|---|
| **HDDA** | 72.0 | 4H x 0.720 | 8H x 0.720 |
| **TMP(EO)6TA** | 23.7 | 6H x 0.237 | 12H x 0.237 |
| **DUA** | 4.3 | 4H x 0.043 | 8H x 0.043 |
| **Total** | 100 | 4.48H | 8.95H |

The amount of AMS and ADT adducts generated during the aza-Michael reaction were estimated on the basis of $^1$H-NMR chemical shift data by the following formulas:

**(1)** $$\% AMS = \frac{I_{e2}}{4.48} x \frac{2.88}{I_{ref}}$$

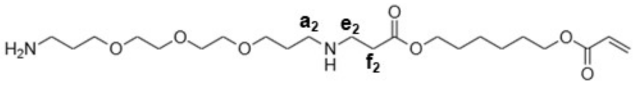

**(2)** $$\% ADT = \frac{I_{e3}}{8.95} x \frac{2.88}{I_{ref}}$$

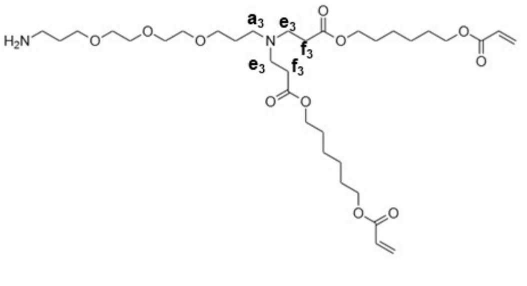

## Fourrier Transformation Infrared Spectroscopy – Attenuated Total Reflexion modus

The $Conv_{C=C}$ was estimated by Fourier Transform Infrared Spectroscopy in Attenuated Total Reflexion modus (FTIR-ATR). The measurement was achieved on Bruker Vertex 70 spectrometer, with a Specac ATR type Quest equipped with a diamond crystal Spectroscopy, with a spectral resolution of 4 $cm^{-1}$ and 30 scans.

During the $AZ_1$ reaction, the decrease of the acrylate band characteristic of $CH=CH_2$ twisting vibration at 810 $cm^{-1}$ was followed taking as a reference a band at 1475 $cm^{-1}$ corresponding to the $CO\text{-}O\text{-}CH_2$ deformation vibration present on each molecule of the formulation. The conversion of acrylate function is calculated according the following equation.

$$Conv_{C=C}\,(\%) = 100\ x\ (1 - \frac{A_t^{810}}{A_0^{810}} * \frac{A_0^{1475}}{A_t^{1475}})$$

The printed objects were analyzed by FTIR-ATR to determine the $Conv_{C=C}$ after printing (hv), after post-curing UV ($PC_{hv}$) and after the occurring of $AZ_2$.

$Conv_{C=C}$ was measured by FTIR-ATR on each side of the printed object (30 x 5 x 0.3 mm). Different values of $Conv_{C=C}$ were observed between the plate side where the first layer was printed and the vat side, corresponding to the last one. This can be explained by a difference of light dose received by each layer. Indeed, the first layer was irradiated with an overcure of 250 µm during to 2.79 seconds to ensure a sufficient adhesion to the moving plate. The next layers were printed with an overcure of 150 µm during 0.81 second. The first, second, third and fourth layers also received an irradiation 3 successive times while the fifth and sixth layers received respectively twice and once irradiation.

For each face of the resulting polymer material the conversion was evaluated after the 3D printing (hv), the post-curing ($PC_{hv}$) and the occurring of $AZ_2$.

**Table S2.** FTIR- ATR measurements of $Conv_{C=C\ on}$ the plate and vat side at the different steps.

| $Conv_{C=C}$ | $AZ_1$ + hv | $AZ_1$ + hv + $PC_{hv}$ | $AZ_1$ + hv + $PC_{hv}$ + $AZ_2$ |
|---|---|---|---|
| Plate side | 74% ±1 | 79% ±1 | 92% ±1 |
| Vat side | 66% ±1 | 71% ±1 | 88% ±1 |
| Average | 70% ±1 | 75% ±1 | 90% ±1 |

## Monitoring of viscosity during aza-Michael addition by Rheometer

The measurement of viscosity was achieved by Rheometer Anton Paar MCR302 with parallel plate (PP), at 20°C, with a constant shear rate of 20/s and an air-gap of 1 mm between the plate and the glass plate. Each point is an average of 30 measurements.

The aza-Michael reaction leads to an evolution of the viscosity:

- During the $AZ_1$, the viscosity increases to approximatively 500 mPa.s between 0 and 120 minutes. The viscosity becomes constant corresponding to the end of $AZ_1$.
- During the $AZ_2$, the viscosity increases because of the creation of additional cross linkers by the formation of the ADT until 7 days.

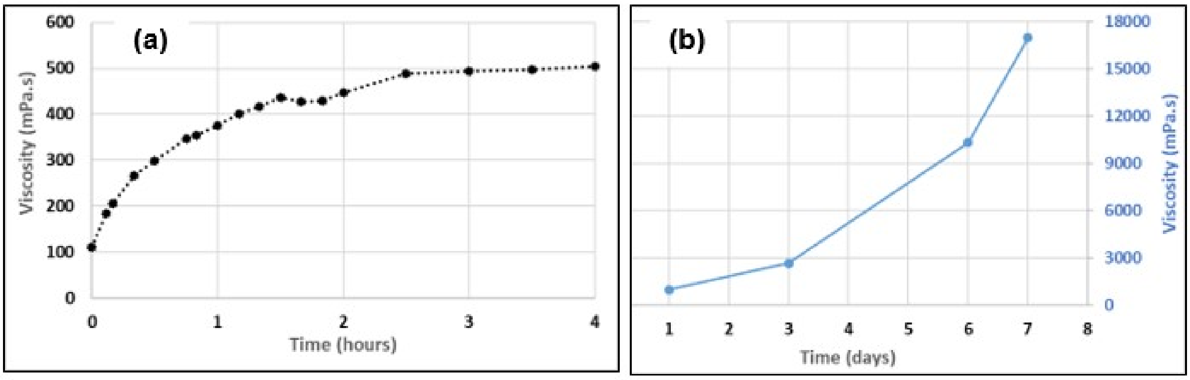


**Figure S2.** Evolution of viscosity during aza-Michael reaction between (a) 0 and 4 hours (b) 1 and 7 days.

## 3D printing and measurements of rectangular printed object

### Determination of Jacobs parameters for the photosensitive resin

The depth penetration of the light (Dp) has to be superior to the layer thickness and inferior to 4 layers to limit the light diffusion and guaranty a good resolution. According to the Jacobs equation, the critical energy, Ec, required to cure the resin to its gel point and the depth penetration Dp of the light were determined and are 1.3 mJ/cm² and 111 µm, respectively.

### 3D printing of the rectangular printed objects

The object (30 x 5 x 0.3 mm) was printed with an Asiga UV Max by additive manufacturing of 6 layers of 50 µm, working at a curing wavelength of 385 nm and an irradiance of 9.8 mW/cm². The first layer was irradiated with an overcure of 250 µm during 2.79 seconds to ensure a sufficient adhesion to the moving plate. The next layers were printed with an overcure of 150 µm during 0.81 seconds.

After the 3D printing, the object was cleaned with ethanol. A post cure under a UV-WAVE LED was achieved on each side of the object at 385 nm and 70 mW/cm², during 2 minutes.

Measurements were made in DMA, with a Q800 of TA Instruments in tensile mode.

3D printed samples (30 x 5 x 0.3 mm) were irradiated at 385 nm with an irradiance of 9.8 mW/cm², during 2 minutes at 70mW/cm² and evaluated after the post cure and after the full process.

### Tensile tests in MultiFrequency-Strain by DMA

A frequency of 1 Hz with an amplitude of 20 µm and a preload force of 0.01N were chosen to ensure the tensile test. The film was analysed between -70°C and 130°C with a temperature ramp of 3°C/min. To characterize the network properties of the polymer formed, the storage modulus (E'), and the Tan δ curve were studied. This analysis gives access to the rubbery modulus ($E_r$), the glass transition temperature (Tg) estimated by the Onset method, the homogeneity of the polymer network with the full width half-maximum (FWMH) of the tgδ curve, and the relaxation temperature (Tr)

### Elongation at break in Controlled Stress mode by DMA

A preload force of 0.001N was chosen to stretch the printed object. The material was heated with a temperature ramp of 5°C/min to the deformation temperature (chosen at the rubbery state by the previous tests in Multi-frequency strain). The curve of elongation at break leads to several parameters:

- The maximal strain ($\varepsilon_{max}$).

- The maximal stress ($\sigma_{max}$).
- The Young modulus, the slope of the curve.

**Evaluation of the SMP properties by thermocycle in Controlled Stress mode by DMA**

The sample was heated above the relaxation temperature (Tr) until the deformation temperature chosen at rubbery state and stretched with a maximal stress before the elongation break ($\varepsilon_{max}$), determinated by the previous tensile tests. Then it is cooled down keeping a constant stress below the Tr, before the increase of the tangent delta. This step induces a stress inside the material and leads to a resulting deformation $\varepsilon_l$. The material is fixed at this temperature removing the stress ($\varepsilon_u$) and recovered with heating above the glass transition temperature in order to reach a final deformation ($\varepsilon_p$).

The fixation ratio depends on the strain obtain at the end of the fixation step ($\varepsilon_u$) and the strain resulting of the cooling step ($\varepsilon_l$) and can be expressed by the following ratio :

$$R_f(N) = \frac{\varepsilon_u}{\varepsilon_l}$$

The recovery ratio is calculated by the following ratio:

$$R_r(N) = \frac{\varepsilon_l - \varepsilon_p(N)}{\varepsilon_l - \varepsilon_p(N-1)}$$

Where $\varepsilon_p(N)$ is the recovered strain at the end of the cycle, after heating the material (in %) and $\varepsilon_p(N-1)$ is the recovered strain at the end of the previous cycle (in %).

**Evaluation of the density of network polymer**

Measurements of the density ($\rho_{polymer}$) of the polymer network were achieve by pyncometry. The parameter was calculated by the following equation.

$$\rho_{polymer} = \frac{m_{polymer}}{v_{pycnometer} - \frac{m_{water}}{\rho_{water}}}$$

Where

$m_{polymer}$ is the weight of the printed object;

$v_{pycnometer}$ the volume of the pycnometer;

$m_{water}$, the weight of the water;

$\rho_{water}$the density of the water.
